# OsGRP3 Enhances Drought Resistance by Altering Phenylpropanoid Biosynthesis Pathway in Rice (*Oryza sativa* L.)

**DOI:** 10.3390/ijms23137045

**Published:** 2022-06-24

**Authors:** Wuwu Xu, Yangfan Dou, Han Geng, Jinmei Fu, Zhiwu Dan, Ting Liang, Mingxing Cheng, Weibo Zhao, Yafei Zeng, Zhongli Hu, Wenchao Huang

**Affiliations:** 1 State Key Laboratory of Hybrid Rice, Wuhan University, Wuhan 430072, China; xuwuwu@whu.edu.cn (W.X.); 2019202040079@whu.edu.cn (Y.D.); genghan@whu.edu.cn (H.G.); 2021202040088@whu.edu.cn (J.F.); zwdan@whu.edu.cn (Z.D.); 2015202040066@whu.edu.cn (T.L.); chengmingxing@whu.edu.cn (M.C.); weibozhao@whu.edu.cn (W.Z.); zengyafei@whu.edu.cn (Y.Z.); huzhongli@whu.edu.cn (Z.H.); 2 College of Life Sciences, Wuhan University, Wuhan 430072, China

**Keywords:** GRP, drought response, phenylpropanoid biosynthesis, lignin, flavonoids

## Abstract

As a sessile organism, rice often faces various kinds of abiotic stresses, such as drought stress. Drought stress seriously harms plant growth and damages crop yield every year. Therefore, it is urgent to elucidate the mechanisms of drought resistance in rice. In this study, we identified a glycine-rich RNA-binding protein, OsGRP3, in rice. Evolutionary analysis showed that it was closely related to OsGR-RBP4, which was involved in various abiotic stresses. The expression of OsGRP3 was shown to be induced by several abiotic stress treatments and phytohormone treatments. Then, the drought tolerance tests of transgenic plants confirmed that OsGRP3 enhanced drought resistance in rice. Meanwhile, the yeast two-hybrid assay, bimolecular luminescence complementation assay and bimolecular fluorescence complementation assay demonstrated that OsGRP3 bound with itself may affect the RNA chaperone function. Subsequently, the RNA-seq analysis, physiological experiments and histochemical staining showed that OsGRP3 influenced the phenylpropanoid biosynthesis pathway and further modulated lignin accumulation. Herein, our findings suggested that OsGRP3 enhanced drought resistance in rice by altering the phenylpropanoid biosynthesis pathway and further increasing lignin accumulation.

## 1. Introduction

Plants always suffer from various kinds of abiotic stresses, such as drought, salinity, heat and cold stress. The stresses impair the growth of plants and cause serious yield loss in global major crops [1,2,3,4]. Among the stresses, drought damages crop production incalculably every year. According to the World Food Security Organization, food production is facing enormous challenges along with global population increasing and water availability decreasing [2]. Therefore, developing drought-resistant varieties of crops is crucial and urgent for food security.

To evade and ameliorate drought-induced damage, plants have developed a variety of strategies through diverse morphological and physiological changes, including stomatal adjustment, osmotic adjustments, antioxidant metabolism and ion regulation [2,5,6]. Some molecular mechanisms underlying drought resistance have been studied extensively, and most have been focused on transcription factors [7,8,9,10,11], kinases [12,13] and the ABA-mediated signaling pathway [1,4,14,15]. Although some RNA-binding proteins have also been reported to be involved in drought response [16,17,18], the mechanisms were less explored.

Glycine-rich RNA binding proteins (GRPs) have been reported to participate in various RNA processes, including mRNA stability, RNA splicing and RNA transport to regulate plant growth and development processes [19]. Magdalena Czolpinska and Michal Rurek have reviewed the structures, classifications and functions of GRPs [20]. GRPs responding to stress have also been studied extensively. For example, *MpGR-RBP1* from *Malus prunifolia* was up-regulated by salinity, oxidation, or abscisic acid [21]. The ectopic expression of *MpGR-RBP1* in *Arabidopsis thaliana* enhanced salt and oxidative stress tolerance. The expression of *OsRBGD3* was induced by cold, drought and salt stress in a drought tolerant rice cultivar [22]. The constitutive overexpression of *OsRBGD3* in *Arabidopsis thaliana* conferred cold stress tolerance and ABA sensitivity. Expressing *NtGR-RBP1* in *E. coli* enhanced salinity, drought, cold and heat shock tolerance [23]. AtGRP7, a well-studied representative GRP, has been reported to be implicated in cold stress response [16], osmotic stress response [16,24], oxidative stress adaption [25], plant immunity [26] and circadian rhythm [27,28,29,30].

Phenylpropanoid metabolism contributes to plant development and plant-environment interactions [31,32,33]. Two major products of the phenylpropanoid biosynthesis pathway, lignin [34,35,36,37] and flavonoids [38], have been reported to positively regulate drought resistance. As a main component of the cell wall, lignin plays an important role in mechanical support and water transport in plants [39,40]. The lignified cell wall helps to control water penetration and transpiration, as well as to maintain cell osmotic balance. Lignin deposition enhances mechanical strength and water impermeability to maintain plant cell turgor, even under water-limiting conditions [41]. Researches have revealed that upregulating the expression of genes involved in lignin biosynthesis, such as phenylalanine ammonia lyase (*PAL*) in *Nicotiana tabacum* [42], *CAD2* and *CAD3* in *Cucumis melo* [43], or promoting the activities of enzymes involved in lignin biosynthesis, such as MePOD and MeCAD15 in *Manihot esculenta* [34], leads to the accumulation of lignin, which helps plants to fight against drought stress. Moreover, overexpressing other genes that were not in the phenylpropanoid pathway, such as *IbLEA14* in *Ipomoea batatas* [44], *PuC3H35* in *Populus ussuriensis* [35], *OsTF1L* and *OsERF71* in rice [45,46], also resulted in the deposition of lignin and enhanced drought tolerance.

Previously, we identified a glycine-rich RNA-binding protein, OsGRP3 (*Os03g0670700*), which interacted with OsDi19-4 through two-hybrid bacteria screening [47]. Since OsDi19-4 was a drought-induced factor, we speculated whether OsGRP3 was also involved in drought resistance. In this study, we found that OsGRP3 bound with itself and responded to various stresses and phytohormones. OsGRP3 regulated phenylpropanoid synthesis and increased lignin accumulation to enhance drought resistance in rice.

## 2. Results

### 2.1. Phylogenetic Analysis of GRPs

**Figure 1:**
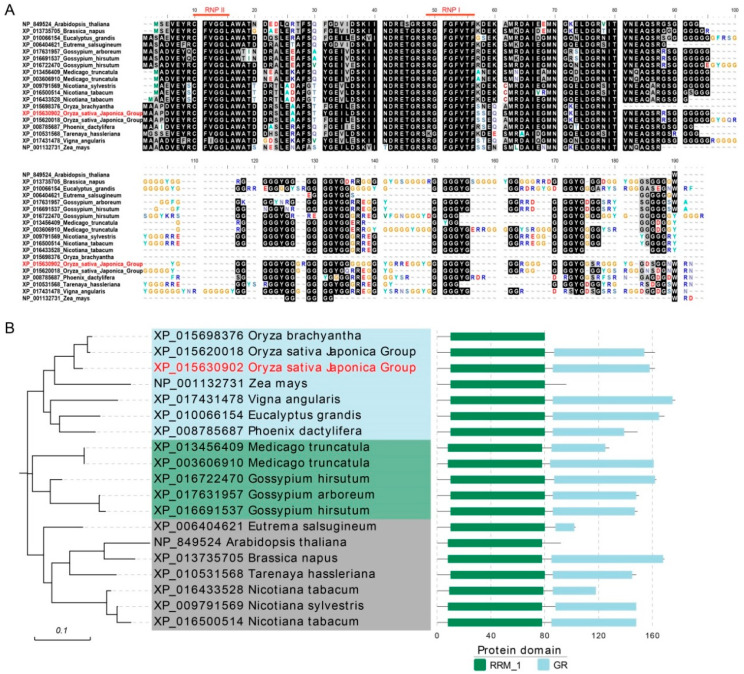
Evolutionary analysis of OsGRP3 in different species. (**A**) multiple sequence alignment of 19 homologous GRPs proteins of different species were aligned by ClustalW and decorated using Bioedit v.7.1.3.0. The red one (XP_015630902) indicates OsGRP3. XP_015620018 indicates OsGR-RBP4. The black background indicates conserved amino acids. RNPI (RGFGFVTF; (K/R)G(F/Y)(G/A)FVX(F/Y)) and RNPII (CFVGGL; (C/I)(F/Y)(V/I)(G/K)(G/N)L) are two RNA-binding consensus sequences. (**B**) Phylogenetic tree of the 19 proteins. Different background colors represent different clades. The corresponding protein structures are shown on the right, and the numbers of amino acids are indicated on the lower coordinate.

OsGRP3 encodes a Glycine-rich RNA binding protein with 162 amino acids. To investigate the evolutionary relationship of OsGRP3 in various species, we downloaded 19 homologous proteins from the Gcorn plant and performed protein multiple sequence alignment. The alignment results showed two conserved atypical motifs embedded in the RNA-recognition motif (RRM) domain, which was composed of approximately 80 amino acids (Figure 1A). The phylogenetic tree indicated that OsGRP3 was the closest to OsGR-RBP4 (Figure 1B). All the homologous proteins comprise the conserved domain RRM, and most of them have a low-complexity region rich in glycine (GR) (Figure 1B).

To investigate the evolutionary relationship of OsGRP3 in rice, we used the protein sequence of OsGRP3 for local blast in rice and extracted 120 homologous protein sequences by TBtools v.1.098696. The corresponding genes were unevenly scattered on 12 chromosomes with the densest genes on chromosome 3 and the sparsest genes on chromosome 12 (Supplementary Figure S1). The phylogenetic tree showed ten clades based on evolutionary study. OsGRP3 was still the closest to OsGR-RBP4 (Supplementary Figure S2). Then, the expression data of these genes were downloaded from the Rice Genome Annotation Project (http://rice.uga.edu/, accessed on 19 August 2020). The heatmap of the expression data indicated that the expression pattern of OsGRP3 was similar to that of OsGR-RBP4. They were both highly expressed in various stages and organizations (Supplementary Figure S3).

Since the expression of *OsGR-RBP4* was reported to be regulated by various abiotic stresses, such as high temperature, low temperature and salt stress [48], we speculated that *OsGRP3* may also be involved in a variety of abiotic stresses.

### 2.2. Expression of OsGRP3 Can Be Induced by Different Hormones and Stress Treatments

**Figure 2:**
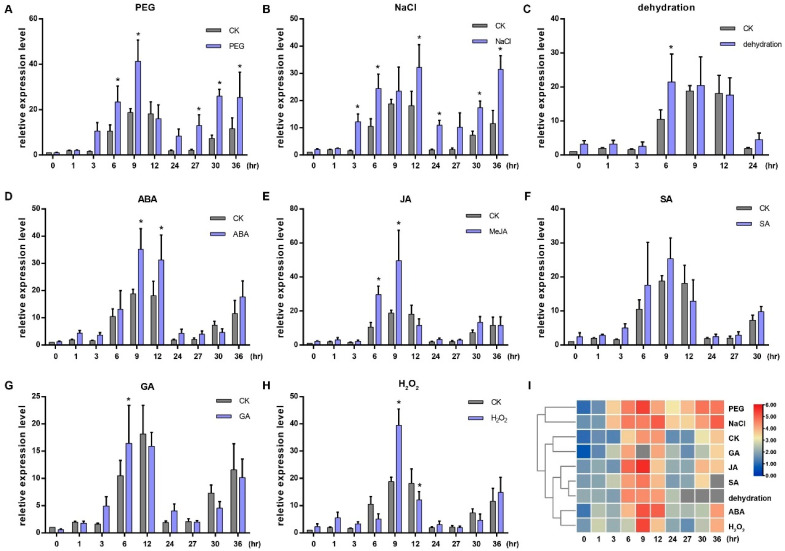
Expressions of *OsGRP3* responding to PEG 6000 (**A**), NaCl (**B**), dehydration (**C**), ABA (**D**), JA (**E**), SA (**F**), GA (**G**) and H_2_O_2_ (**H**). CK indicates control. All the relative expression values of *OsGRP3* under different treatments at different times were calculated compared with the expression value of CK at 0 h, which was normalized to 1. The significant differences were determined using the Student’s *t*-test, * *p* < 0.05. The horizontal axis shows the processing time. (**I**) Cluster analysis of the expressions of OsGRP3 responding to different treatments.

We investigated the effects of abscisic acid (ABA), methyl jasmonate (MeJA), salicylic acid (SA), gibberellic acid (GA), PEG6000, NaCl, H_2_O_2_ and dehydration treatments on the expression of *OsGRP3* at 0, 1, 3, 6, 9, 12, 24, 27, 30 and 36 h after treatments via quantitative real-time PCR (qRT-PCR). The results indicated that the expression of *OsGRP3* was periodic, and it was significantly affected by all the treatments except SA (Figure 2A–H). Both the PEG6000 and NaCl induced the expression of *OsGRP3* at several time points (Figure 2A,B). The cluster analysis of rows in the expression heatmap showed that PEG6000 and NaCl treatments had the largest difference compared with normal conditions (Figure 2I). All of this indicated that *OsGRP3* was involved in various stresses and hormone responses, especially salt and drought stress responses.

### 2.3. OsGRP3 Enhanced Drought Resistance in Rice

As the expression of *OsGRP3* was significantly induced by PEG6000, we generated transgenic plants to investigate how *OsGRP3* responds to drought stress. Three overexpression (OE) lines, OE-91, OE-109 and OE-127, were obtained, as well as three knockout (KO) lines, *Osgrp3-27*, *Osgrp3-52* and *Osgrp3-59*. The mutant sites are shown in Supplementary Figure S4.

**Figure 3:**
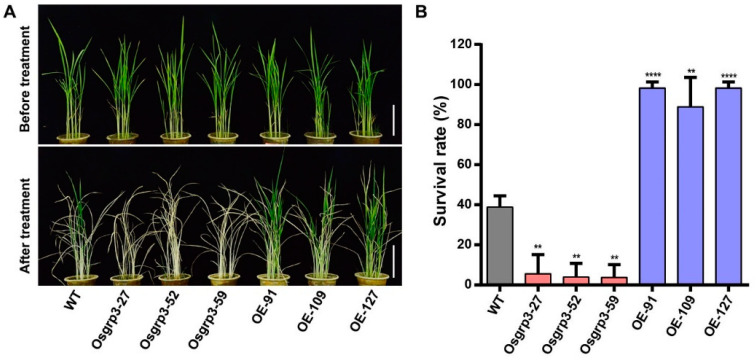
Drought resistance detection of *OsGRP3* transgenic materials. (**A**) Phenotypic characterization of knockout lines, OE lines and WT under drought treatment. The scale bars indicate 10 cm. (**B**) Survival rates of the transgenic lines and WT under drought treatment. The significant differences were determined using the Student’s *t*-test, *p* < 0.05. **, *p* < 0.01; ****, *p* < 0.0001.

Then, the 21-day-old seedlings of transgenic plants and wild-type ZH11 (WT) grown in soil were drought-treated for two weeks and then re-watered for 9 days. When compared with the WT, the OE lines developed better and had greater survival rates under stress conditions, whereas the KO lines showed lower survival rates (Figure 3A,B). These results indicated that OsGRP3 enhanced drought resistance in rice.

### 2.4. OsGRP3 Formed Homodimers or Homomultimers

**Figure 4:**
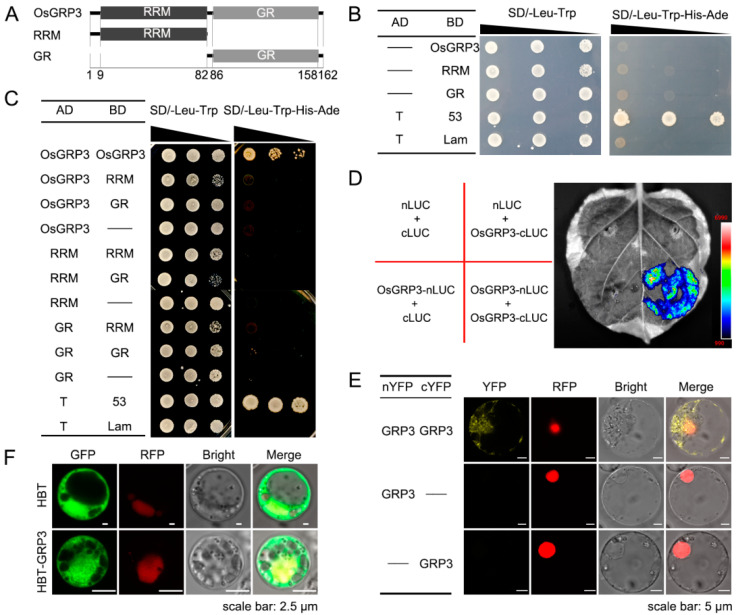
Autoassociation of OsGRP3. (**A**) The OsGRP3 was truncated as two parts RRM and GR according to the number of amino acids shown on the axes. (**B**) Autoactivation test of full length of OsGRP3 and truncated parts, RRM and GR, by yeast two-hybrid assays. “__” indicates empty victor. T and 53 were co-transferred as positive controls, and T and Lam as negative controls. (**C**–**E**) Autoassociation test of OsGRP3. (**C**) Yeast two-hybrid assays. The annotations in (**C**) are the same as in (**B**). (**D**) Bimolecular luminescence complementation assay. The texts on the left in (**D**) indicate the different combinations of carrier strains injected into the right leaf. (**E**) Bimolecular fluorescence complementation assay. The scale bars indicate 5 μm. (**F**) Subcellular localization of OsGRP3. The scale bars indicate 2.5 μm. bZip63-RFP is used as nuclear markers in (**E**,**F**).

Self-association of some GRPs were reported to affect their functions involved in the RNA process [49,50]. Herein, we performed yeast two-hybrid (Y2H) assay to test whether OsGRP3 could bind with itself. First, we used AlphaFold2 to predict the protein structure of OsGRP3 and its self-association. The RRM domain of OsGRP3 was predicted to consists of two α-helices and a 4-strand β-sheet like other typical RRM domains that have been validated before [51,52]. However, the C-terminal region showed disordered without a fixed configuration (Supplementary Figure S5A). The RRM domain was predicted to be responsible for the self-binding of OsGRP3 (Supplementary Figure S5B). According to the predicted results, OsGRP3 was truncated into two parts, RRM and GR (Figure 4A). Then, the transcriptional autoactivation of OsGRP3, RRM and GR was detected. The results showed that all of them had no transcription activity (Figure 4B). Then, Y2H assay was carried out to detect whether it could form homodimers or homomultimers. The result showed that OsGRP3 could only interact with itself of full length, not with the truncated protein, RRM or GR (Figure 4C). Additionally, bimolecular luminescence complementation (BiLC) assay using a split luciferase (LUC) system and bimolecular fluorescence complementation (BiFC) assay using a split yellow fluorescent protein (YFP) system also confirmed this interaction in the *N. benthamiana* leaf and rice protoplasts (Figure 4D,E). The preceding evidence strongly demonstrated that OsGRP3 could bind with itself. Subsequently, subcellular localization exhibited that OsGRP3 localized both in the nucleus and cytoplasm (Figure 4F). This may be related to the properties of GRPs for nucleoplasmic shuttling and mRNA transporting.

### 2.5. Transcriptome Profiling of Drought Response in Rice Seedlings

**Figure 5:**
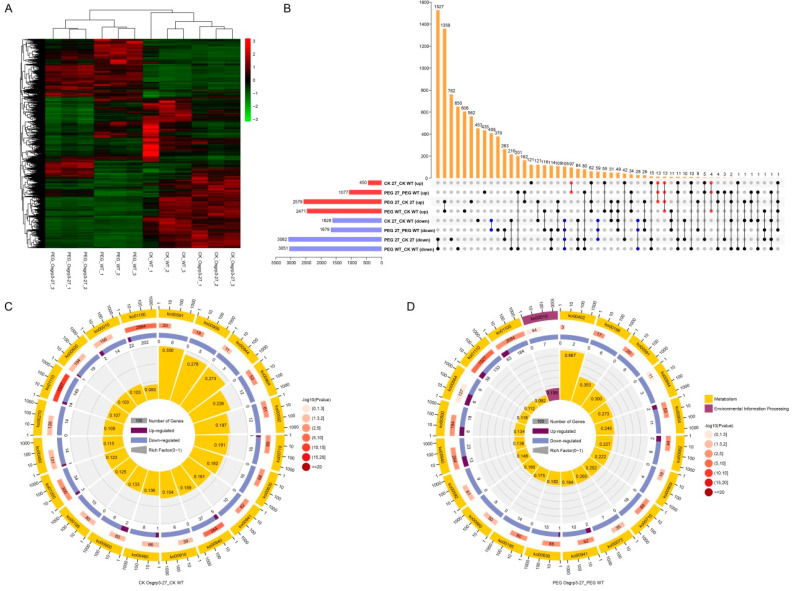
Transcriptome analysis of *Osgrp3-27* and WT under control and PEG6000 treatment. (**A**) Cluster analysis of all differentially expressed genes (DEGs). (**B**) Venn plots of DEGs in wild-type ZH11 and *Osgrp3-27* under control and 20% PEG6000 treatment. Red represents up-regulated DEGs, and blue represents down-regulated DEGs. Orange represents the intersection of different combinations that solid points refer to. (**C**,**D**) KEGG analysis of DEGs between *Osgrp3-27* and WT under control (**C**) and under PEG6000 treatment (**D**). Numbers on a red background indicate all genes with the corresponding pathway annotation. Numbers on purple and blue backgrounds indicate up-regulated and down-regulated genes separately. The ko number stands for the KEGG pathway.

To investigate how OsGRP3 influenced the transcription of other genes, we chose the KO line *Osgrp3-27* and WT for RNA-seq under normal conditions and 20% (*w*/*v*) PEG6000 treatments. The heat map of differentially expressed genes (DEGs) showed that the results of sequencing were repeatable, and the expression levels of numerous genes were changed between WT and *Osgrp3-27* under both conditions (Figure 5A). There were 450 genes up-regulated and 1628 genes down-regulated in *Osgrp3-27* compared with WT under normal conditions, while 1077 genes were up-regulated and 1679 were down-regulated in *Osgrp3-27* under PEG6000 treatment. Furthermore, 127 genes were up-regulated in *Osgrp3-27* under both normal conditions and PEG treatment, while 601 genes were down-regulated (Figure 5B).

KEGG pathway enrichment analysis showed that DEGs were most significantly enriched in the biosynthesis of secondary metabolites (ko01110), metabolic pathways (ko01100) and phenylpropanoid biosynthesis (ko00940) under normal conditions (Figure 5C). Under PEG6000 treatment, the DEGs were most significantly enriched in the biosynthesis of secondary metabolites, carbon fixation in photosynthetic organisms (ko00710) and diterpenoid biosynthesis metabolic pathways (ko00904) (Figure 5D). Apart from these, the biosynthesis of secondary metabolites, metabolic pathways, phenylpropanoid biosynthesis, carbon fixation in photosynthetic organisms, diterpenoid biosynthesis, glyoxylate and dicarboxylate metabolism (ko00630), linoleic acid metabolism (ko00591) and flavonoid biosynthesis (ko00941) were all obviously changed in *Osgrp3-27* compared with WT under both normal conditiona and PEG6000 treatment.

### 2.6. OsGRP3 Regulated Phenylpropanoid Biosynthesis and Enhanced Lignin Accumulation in Rice

**Figure 6:**
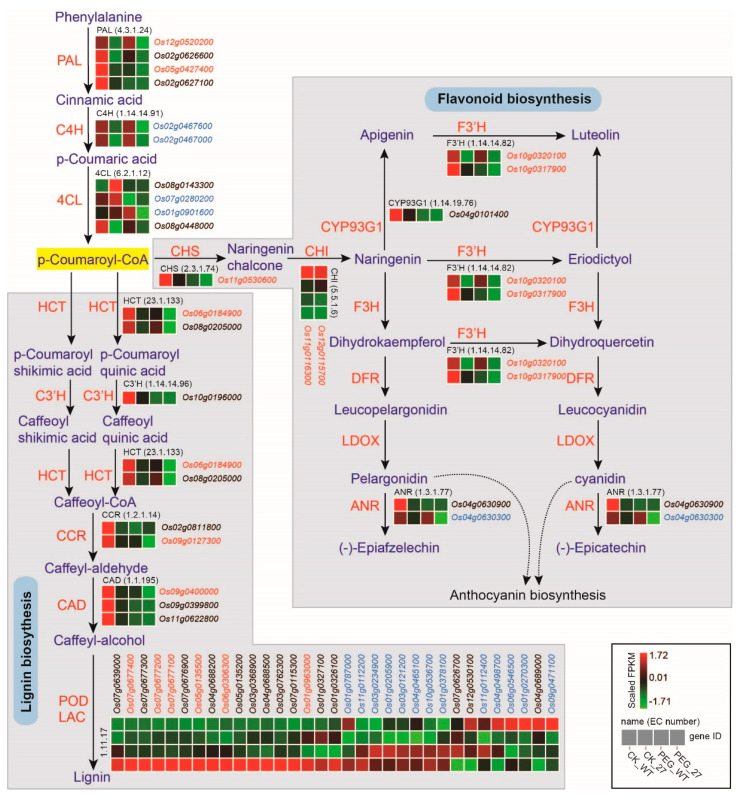
Differentially expressed genes in phenylpropane biosynthesis pathway including lignin biosynthesis and flavonoid biosynthesis. DEGs under normal conditions are shown in black. DEGs under PEG6000 treatment are shown in blue. DEGs under both conditions are shown in red. The three initial steps of phenylpropane metabolic pathway were catalyzed by phenylalanine ammonia lyase (PAL), cinnamate 4−hydroxylase (C4H) and 4−coumarate−CoA ligase (4CL). The flavonoid biosynthesis was catalyzed by chalcone synthase (CHS), chalcone isomerase (CHI), flavanone 3−hydroxylase (F3H), flavonoid 3′−hydroxylase (F′3H), flavone synthase II (CYP93G1), dihydroflavonol 4−reductase (DFR), anthocyanidin synthase (ANS/LDOX) and anthocyanidin reductase (ANR). The lignin biosynthesis was catalyzed by shikimate O−hydroxycinnamoyltransferase (HCT), 5−O−(4−coumaroyl)−D−quinate 3′−monooxygenase (C3′H), cinnamoyl−CoA reductase (CCR), cinnamyl alcohol dehydrogenase (CAD) and peroxidase (POD).

The phenylpropanoid biosynthesis pathway has been reported to participate in various life processes during plant growth [31]. Additionally, two major metabolites of the pathway, lignin and flavonoid, have been reported to positively regulate drought resistance [34,35,36,37,38]. As the KEGG analysis showed that DEGs were significantly enriched in phenylpropanoid biosynthesis and flavonoid biosynthesis under both conditions, we investigated which steps of the pathway these DEGs were distributed in. The transcriptome profiling indicated that DEGs enriched in the flavonoid biosynthesis pathway were mainly annotated as chalcone synthase (CHS), chalcone isomerase (CHI), flavonoid 3′-hydroxylase (F3′H) and anthocyanidin reductase (ANR). DEGs involved in lignin biosynthesis were distributed in nearly all the biosynthesis steps, especially the last step catalyzed by peroxidases (PODs) (Figure 6). This showed that most of the DEGs were down-regulated in the lignin biosynthesis pathway.

**Figure 7:**
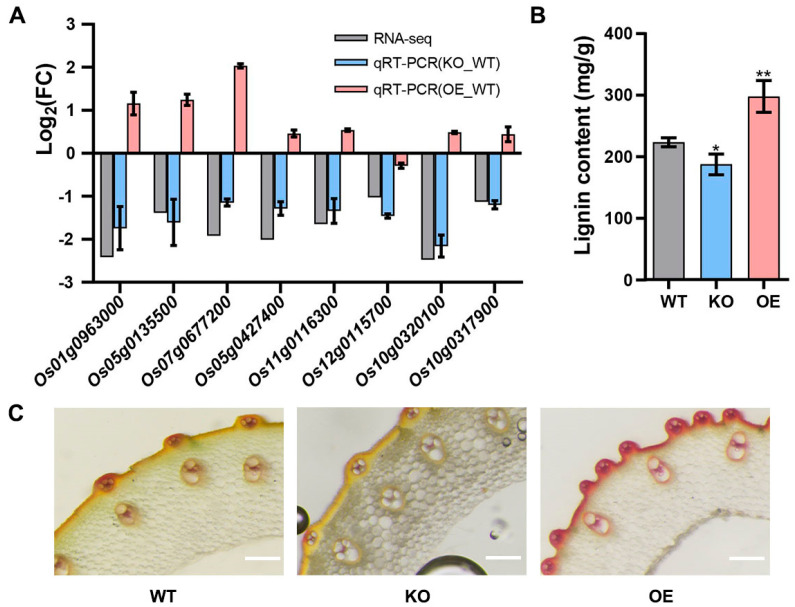
Detection of lignin content. (**A**) qRT−PCR of common DEGs in phenylpropane biosynthesis pathway. (**B**) Detection of lignin content. The significant differences were determined using the Student’s *t*-test, *p* < 0.05. *, *p* < 0.05; **, *p* < 0.01. (**C**) Cross−sections of the stems of WT, *Osgrp3−27* (KO) and OE−127 (OE), with phloroglucinol staining to detected lignin content. The scale bars indicate 200 μm.

Then, we randomly selected eight common DEGs in the phenylpropanoid biosynthesis pathway under both conditions to detect their expression using qRT-PCR. The results showed that these eight genes were down-regulated in *Osgrp3-27* compared with WT, as shown in the transcriptome profiling, while most of them were up-regulated in OE-127 compared with WT (Figure 7A). As most DEGs were enriched in lignin biosynthesis, we further detected the lignin content and carried out a stem section staining experiment of transgenic lines and WT. The results showed that the lignin content was significantly lower in *Osgrp3-27* and higher in OE-127 than that in the WT (Figure 7B,C). All of these demonstrated that OsGRP3 participated in the phenylpropanoid biosynthesis pathway and further regulated the synthesis of lignin.

## 3. Discussion

Plants have developed various mechanisms to respond to drought stress, which is one of the major natural disasters that usually damages crop development and productivity seriously. Therefore, it is important to elucidate the mechanism of drought resistance in plants to develop drought-resistant cultivars. Some studies have shown that GRPs are involved in drought response. It has been reported that expressing *AtGRP2* or *AtGRP7* in rice enhances drought resistance and increases grain yields under drought conditions [17], while overexpressing *AtGRP7* in *Arabidopsis thaliana* delays the germination and seedling growth under dehydration stress conditions [16]. In addition, *atRZ-1a* also negatively regulates the germination and seedling growth of *Arabidopsis thaliana* under dehydration stress conditions, and its expression is suppressed by dehydration [53,54]. Additionally, *SCRGP-1* in *Solanum commersonii* was reported to be induced by drought [55]. In this study, drought tolerant tests demonstrated that OsGRP3 enhanced drought resistance. The overexpression lines showed more resistant to drought compared with the WT, and the knockout lines showed the opposite. The phenotype was consistent with that reported by Jae et.al [56]. This indicated that the phenotype identification was credible and OsGRP3 does have a positive effect on improving drought resistance in rice.

The self-association of GRPs may influence their function involved in RNA processing, for instance, the self-association of TDP-43 and FUS, contributing to RNA granule formation and/or pathologic aggregates in vivo [50]. The self-association of NtGR-RBP1 resulted in the cooperative unfolding of non-native substrate RNA structures, thereby enhancing its chaperone function [49]. In our study, OsGRP3 was identified to bind with itself through Y2H, BiFC and BiLC assay. As OsGRP3 has been reported to have RNA chaperone activity [57], we speculated that the self-association of OsGRP3 may also influence its chaperone function like NtGR-RBP1. Jae et.al suggested that OsGRP3 dually regulated the stability of its different target mRNAs. This may result from the interaction between OsGRP3 and its partners in P-bodies [56]. This means that OsGRP3 may also interact with other proteins. In fact, many GRPs have been reported to interact with themselves or other proteins. Apart from the two examples above, ORRM4 in *Arabidopsis thaliana* was proven to form homodimers with itself and heterodimers with ORRM3 [58]. ORRM3 was also found to bind with itself and form heterodimers with ORRM2 [59]. The ORRM5 was proven to interact with ORRM3 and ORRM4 but not itself [60]. RBP-P in rice endosperm cells can interact with itself, RBP-L and RBP-208 [61]. The binding of GRPs to themselves or to other proteins may explain the differences of their functions, such as the propensity to bind RNA under different conditions, and the positive or negative regulation of RNA stability. Some researchers have reported that GRPs can also interact with enzymes [62]. This means that GRPs may not only have the chaperone function but also be directly involved in metabolic regulation. The function of dimerization or multimerization of GRPs requires further exploration. Additionally, the partners that GRPs interacted with require further screening.

In this article, we found that OsGRP3 was localized in the nucleus and cytoplasm. A previous study reported that AtGRP7 was localized in both the nucleus and cytoplasm, and participated in the export of mRNAs from the nucleus to the cytoplasm under cold stress conditions [16]. AtJAC1, a JACALIN-LECTIN protein, interacted with AtGRP7 to influence the nucleocytoplasmic distribution of AtGRP7 [29]. TaVER2, another jacalin lectin in wheat, interacted with TaGRP2 to reduce the nuclear accumulation of TaGRP2 [63]. Both of the two interactions further influenced the RNA process of GRPs targets. Therefore, there may also be some proteins that interact with OsGRP3 and change its nucleocytoplasmic distribution to regulate the RNA process of its targets. The jacalin lectin proteins in rice can be further screened and validated as candidate interacting proteins of OsGRP3. Meanwhile, how the nucleocytoplasmic distribution of OsGRP3 influences the RNA process or other biological processes requires more exploration.

It was reported that AtGRP9, in response to salt stress, interacted with AtCAD5, which was involved in lignin synthesis [62]. As AtCAD5 was a key enzyme in the lignin biosynthesis pathway, the author suggested that AtGRP9 affects lignin synthesis by interacting with AtCAD5 to respond to salt stress [64]. In our study, the RNA-seq and qRT-PCR showed that a many of the POD enzymes catalyzing lignin biosynthesis were down-regulated in the *Osgrp3* mutant line. The lignin content of *Osgrp3-27* was lower than that of the wild type, while it exhibited the opposite result in the OE line. This showed that the *Osgrp3* mutant reduced lignin accumulation with a low expression of the *PODs*. Jae et.al reported that OsGRP3 positively regulated drought resistance by reducing reactive oxygen species, which can be scavenged by POD enzymes [56]. They detected that the contents of H_2_O_2_ were up-regulated in *Osgrp3* mutants and down-regulated in OE lines. Combined with the research of Jae et al., we suggested that the decrease in POD enzyme content led to the decrease in lignin content and the increase in H_2_O_2_ content in the *Osgrp3* knockout line. Therefore, OsGRP3 positively regulated lignin biosynthesis and H_2_O_2_ scavenging by PODs to enhance drought resistance in rice.

## 4. Materials and Methods

### 4.1. Plant Materials, Hormones and Stress Treatments

The rice (*Oryza sativa* L.) variety Zhonghua 11 (ZH11) was grown in Yoshida solution in a greenhouse at 28 °C under a 12 h light/12 h dark photoperiod with relative humidity of around 70%. The leaf tissue of the two-week-old seedlings were harvested at 0, 1, 3, 6, 9, 12, 24, 27, 30 and 36 h after treatment with 20% (*w*/*v*) PEG6000 solution, 150 mM NaCl, 100 μM abscisic acid (ABA), 100 μM methyl jasmonate (MeJA), 100 μM salicylic acid (SA), 100 μM gibberellic acid (GA) and 1 mM H_2_O_2_. Then, the expressions of *OsGRP3* were detected using qRT-PCR. The seedlings of transgenic lines and WT were grown in soil for 3 weeks, then not watered for 2 weeks and rewatered for 9 days. The survival rates were counted.

### 4.2. Phylogenetic Analysis

Homology sequences of OsGRP3 were retrieved from a database of plant gene phylogeny, Gcorn Plant (http://www.plant.osakafu-u.ac.jp/~kagiana/gcorn/p/19/, accessed on 21 May 2019). Then, these sequences were aligned using ClustalW in MEGA v.7.0.26 [65]. The phylogenetic tree was constructed through the neighbor-joining method, and the evolutionary distances were produced with MEGA v.7.0.26 using bootstrap analysis (1000 replicates). Additionally, the tree was decorated by Evolview (https://www.evolgenius.info/evolview/#login, accessed on 24 May 2019).

### 4.3. Yeast Two-Hybrid Assays

The coding sequence of *OsGRP3* was cloned into *pGADT7* and *pGBKT7* vectors separately. Then, the constructs were co-transformed into yeast strain AH109. The transformed yeast was plated on a synthetic complete medium lacking Trp and Leu. After being left to grow for 3 days, the clones were transferred to medium lacking Trp, Leu, His and Ade for 3–5 days to test protein interactions.

### 4.4. Bimolecular Luminescence Complementation Assay

The coding sequence of *OsGRP3* was cloned into vector *JW771* (*NLUC*) and *JW772* (*CLUC*). Then, the constructs were transformed into the *Agrobacterium tumefacien* GV3101 (pSoup-p19) competent cell. The 4-week-old *Nicotiana benthamiana* leaves were injected with different combinations of the constructs for BiLC analyses and grew for 48 h under a 12 h light/12 h dark period. Then, the leaves were injected with 1 mM luciferin, and the luciferase signals were detected and captured using the Tanon-5200 image system (Tanon, Shanghai, China). The experiments were repeated at least three times, and yielded similar results.

### 4.5. Bimolecular Fluorescence Complementation Assay

The coding sequence of *OsGRP3* was cloned into vectors *pSPYNE* and *pSPYCE*. Additionally, the constructs were co-transferred into rice protoplasts. The fluorescence of the yellow fluorescent protein was observed and imaged under a confocal laser scanning microscope (Laica, Mannheim, Germany) using 510 nm laser excitation (5% power), a ×63 oil immersion lens and a wavelength detection window of 520–580 nm.

### 4.6. Subcellular Localization

The coding sequence of *OsGRP3* was cloned into the *pHBT* vector. Additionally, the constructs were transferred into rice protoplasts, which were isolated and transfected according to the protocol in [66]. The fluorescence of green fluorescent protein (GFP) was observed and imaged under a confocal laser scanning microscope (Laica, Mannheim, Germany) using 488 nm laser excitation (5% power), a ×63 oil immersion lens and a wavelength detection window of 500–550 nm.

### 4.7. RNA Extraction, cDNA Synthesis and qRT- PCR

Total RNA was extracted using the Trizol reagent (Invitrogen, Carlsbad, CA, USA) according to the instruction manual. Complementary DNA was synthesized using HiScript II reverse transcriptase (Vazyme, Nanjing, China) according to the manufacturer’s instructions. qRT-PCR was performed in triplicate on the CFX96 Touch™ Real-Time PCR Detection System (Bio-Rad, Hercules, CA, USA) with the Hieff qPCR SYBR Green Master Mix (Yeasen, Shanghai, China) according to the manufacturer’s instructions. Expression analysis was performed with three biological replicates. All primers used for gene expression analysis are listed in Supplementary Table S1. The heatmap was generated using TBtools v.1.098696 with rows clustered [67].

### 4.8. Generation of Transgenic Plants

The coding sequence of *OsGRP3* was cloned and inserted into the vector *pCAMBIA1301* under the control of the *UBI* promoter using a one-step clone kit (Vazyme, Nanjing, China) for the overexpression of *OsGRP3*. The vector for knockout lines was constructed referring to the CRISPR/Cas9 System [68]. The plasmids were extracted using the TIANprep mini plasmid kit (TIANGEN, Beijing, China) and sent to BIOGLE GeneTech (Changzhou, Jiangsu, China) to generate the transgenic plant.

### 4.9. RNA Sequencing (RNA-seq) and Data Analysis

The WT and *Osgrp3-27* lines were planted in Yoshida solution for two weeks, as described above, and then half of them were treated with 20% PEG6000 for six hours. The total RNA was extracted as described above from simples, which were fully ground in liquid nitrogen, and thoroughly mixed with 6 complete individuals growing under normal condition and PEG6000 treatment. Three biological replicates were performed. The mRNA libraries were constructed and sequenced using Seqhealth (Wuhan, Hubei, China) with the Hiseq-PE150 (Illumina, San Diego, CA, USA) high-throughput sequencing platform. The clean reads were mapped to the Nipponbare reference genome (http://rice.plantbiology.msu.edu, accessed on 23 January 2021). The DEGs between samples were defined using a cut-off change of at least a 2-fold change (|FC| ≥ 2.0) and *p* value < 0.05. KEGG analysis wasperformed using the OmicShare tool, a free online platform for data analysis (https://www.omicshare.com/tools, accessed on 9 July 2021).

### 4.10. Lignin Content Detection

The lignin content of the second stem above ground of mature rice was determined using the Lignin Content Assay Kit (Biobox, Beijing, China) according to the instructions. The experiments were repeated at least three times.

### 4.11. Histochemical Staining

Vibratome cross-sections (150 μm) of WT, *Osgrp3-27* and OE-127 were taken from the first stem below the panicle at 10 cm above the stem node, and then stained with phloroglucinol-HCl solution (5% in ethanol: water (95:5, *v*/*v*); 36% HCl) for 5 min to distinguish the lignified cell walls and imaged using a Leica DMi8 equipped with a DMC6200 camera (Leica, Mannheim, Germany)

### 4.12. Statistical Analysis

All experiments in this study were carried out with at least three biological replicates. Significant differences were determined using the Student’s *t*-test at *p* < 0.05.

## 5. Conclusions

In this study, we demonstrated that various stresses and hormone treatments could induce the expression of OsGRP3, which could form homodimers or homomultimers with itself like some other GRPs. OsGRP3 enhanced drought resistance in rice by altering the phenylpropanoid pathway and further promoting lignin accumulation.

## Data Availability

The sequences of genes and proteins mentioned in our study are available for download from the public database mentioned above.

## Supplementary Materials

The following supporting information can be downloaded at: https://www.mdpi.com/article/10.3390/ijms23137045/s1.
