# Supplementary material for: OsGRP3 Enhances Drought Resistance by Altering Phenylpropanoid Biosynthesis Pathway in Rice (Oryza sativa L.)
Source: Int J Mol Sci. 2022 Jun 24;23(13):7045. doi: 10.3390/ijms23137045 (PMC9266740; doi:10.3390/ijms23137045)
Supplement: Supplementary file 1 [file ijms-23-07045-s001.zip › ijms-1754256-supplementary.pdf]

Supplementary Materials

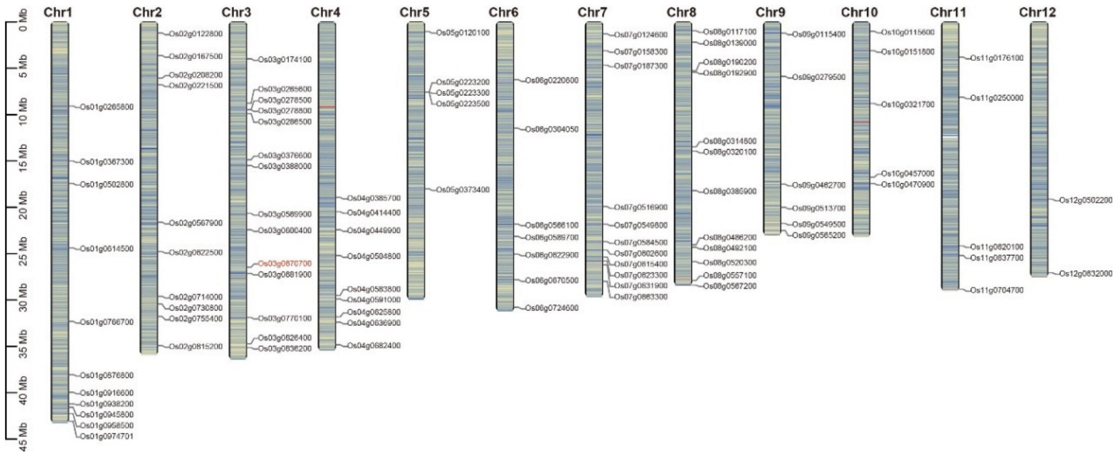

Figure S1. Chromosome distribution of *OsGRP3* homologous genes in rice. The red text indicated *OsGRP3*.

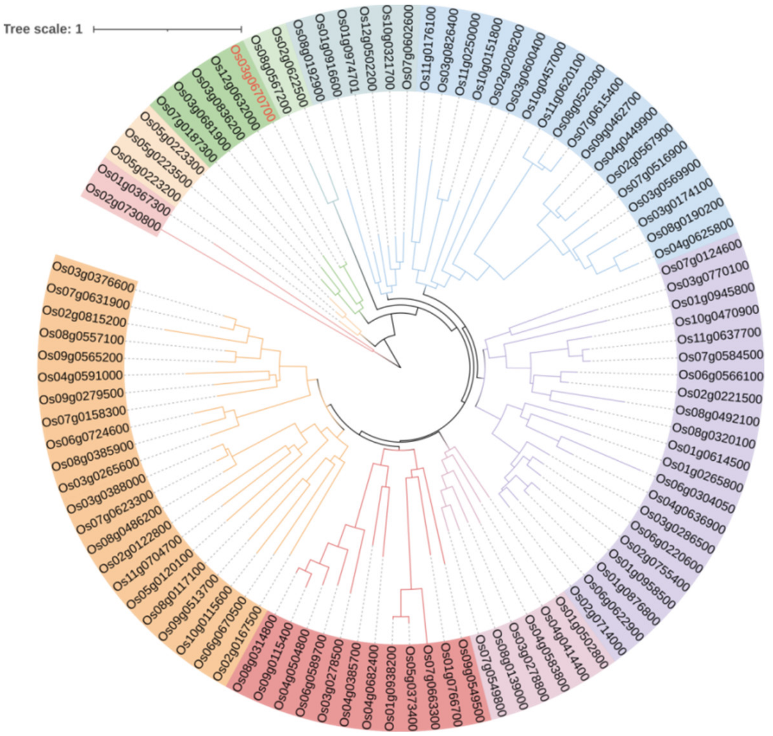

Figure S2. Phylogenetic tree of OsGRP3 homologous proteins in rice. Sequences are multiplely aligned by ClustalW. And phylogenetic reconstruction was generated using the neighbor-joining (NJ) clustering method with 1000 replicates of Bootstrap numbers. Different color of circles represents different clades. The red one indicated OsGRP3.

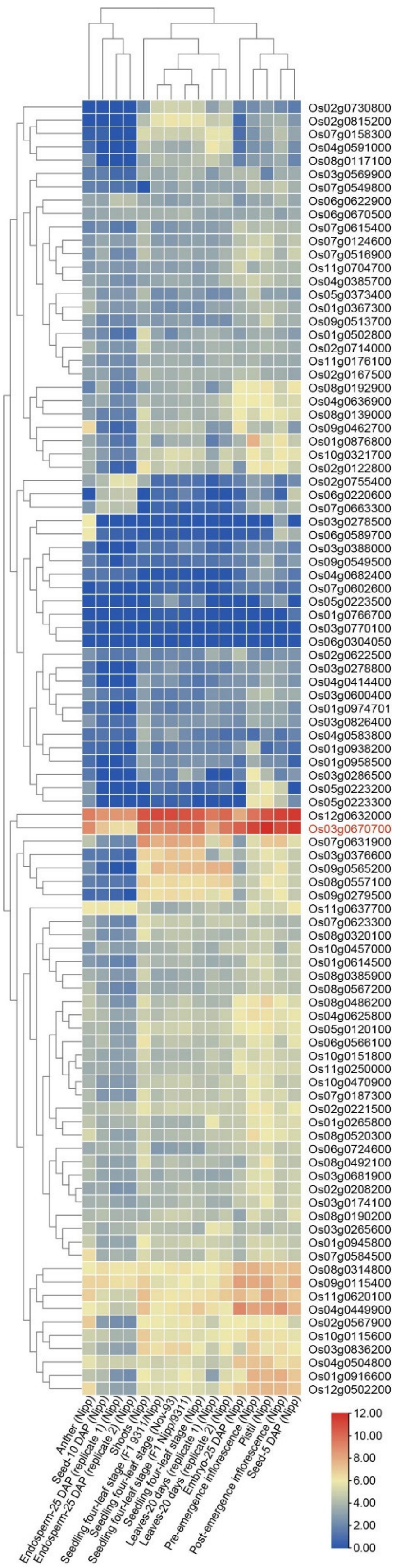

Figure S3. Expression profile of *OsGRP3* homologous genes in rice. The red text indicated *OsGRP3*. DAP: Days after pollination. Nipp: Nipponbare. F1: Hybrid F1.

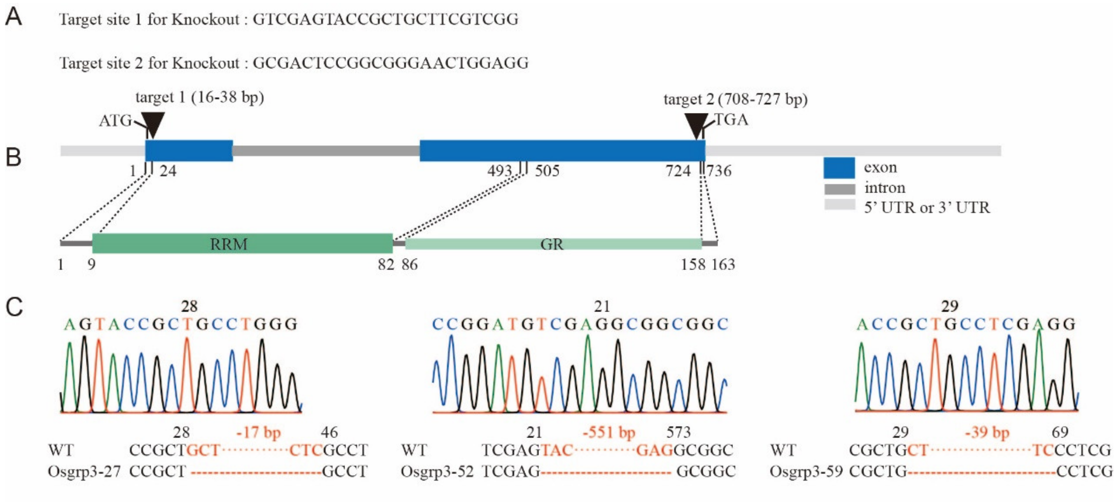

Figure S4. Identification of knockout lines of *OsGRP3*. (**A**) Target sites for knockout lines using crispr/cas9 system. (**B**) Schematic diagram of OsGRP3 gene structure and protein structure. The gene structure is shown above and the protein structure is shown below. Numbers indicate the corresponding nucleic acid bases or amino acids. (**C**) Mutation sites of the knockout lines.

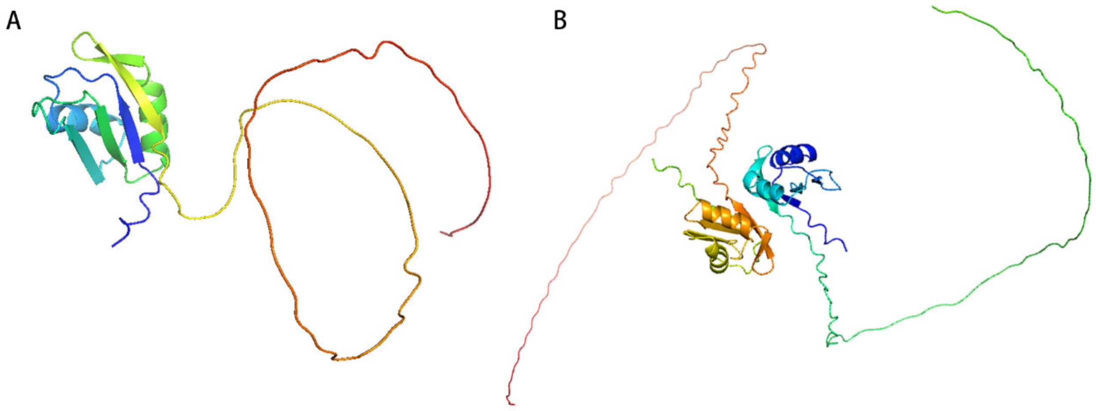

Figure S5. Structure (**A**) and Auto-association (**B**) of OsGRP3 predicted by AlphaFold2.

Supplementary Table S1. List of oligonucleotide primers used in this study.

Application	Name	Forward (+)	Reverse (-)
qRT-PCR	qOsGRP3	CCTTTTCCTCCTCGGTTCGG	CCCGTCTCCCTGTCGTTGAT
qRT-PCR	Os01g0963000	GTGGTACGATGTTCCCCTCG	GTCGACTTGAGGGAAGAGGC
qRT-PCR	Os05g0135500	GGGACAACAAGGCGAACAAC	CAGCAAGGTAGGCGCTATCA
qRT-PCR	Os07g0677200	CTCGGCGACGTTCTATGACA	CGATGACGCTGAATCCCCTC
qRT-PCR	Os05g0427400	GACCACCTCACCCACAAGC	TCCTGCTTCGGCTTCATCAG
qRT-PCR	Os11g0116300	ATGTACTGTGCAGTGGGCA	ACCCCGATGGCGTTGTATTT
qRT-PCR	Os12g0115700	ATGTACTGTGCAGTGGGCA	ACCCCGATGGCGTTGTATTT
qRT-PCR	Os10g0320100	GCGCTACCCTCCTAGTCAAC	AGCGCATATTCTCCGTCCTG
qRT-PCR	Os10g0317900	CAGGATCACCGACACGGAAA	GCCTATCACGACCCACAACA
qRT-PCR	Actin	CCTCTTCCAGCCTTCCTTCATAG	CGATGTTGCCATATAGATCCTTCC
JW771/ JW772	GRP3-LUC	GAGCTCGGTACCCGG-GATCCATGGCGGCGCCGGATGT	GCGTACGAGATCTGGTCGAC-GTTCCTCCAGTTCCCGCCGG
pSPYNE/pSPYCE	GRP3-YFP	TGGCGCGCCACTAGTG-GATCCATGGCGGCGCCGGATGT	GACAGTACTA-TCGATGGATCCGTTCCTCCAG-TTCCCGCCGG
pHBT	OsGRP3-GFP	TCTCCCCTTGCTCCGTG-GATCCATGGCGGCGCCGGATGT	CGCCCTTGCTCAC-CATGGATCCGTTCCTCCAGTTCCCGCCGG
pGADT7	AD-GRP3	GCTCATATGGCCATGGAGGCCAG-TGAATTCATGGCGGCGCCGGATGT	ATTCATCTG-CAGCTCGAGCTCGATGGATCCTCAG-TTCCTCCAGTTCC
pGBKT7	BD-GRP3	TGCAT-ATGGCCATGGAGGCCGAATTCATGGCG GCGCCGGATGT	CAAGGGGTTATGCTAG-TTATGCGGCCGCTCAGTTCCTCCAGTTCC

pGADT7	AD-RRM	GCTCATATGGCCATGGAGGCCAG-TGAATTCATGGCGGCGCCG-GATGTCGAGTACC	ATTCATCTG-CAGCTCGAGCTCGATGGATCCTCAC-TGGGCCTCATTGACGGTGATGTTG
pGBKT7	BD-RRM	TGCAT-ATGGCCATGGAGGCCGAATTCATGGCGGCGCCGGATGTCGAGTACC	CAAGGGGTTATGCTAG-TTATGCGGCCGCCTGGGCCTCATTGAC-GGTGATGTTG
pGADT7	AD-GR	GCTCATATGGCCATGGAGGCCAG-TGAATTCTCCCGCCGCTCCGGCGGCG-GAGGCG	ATTCATCTG-CAGCTCGAGCTCGATGGATCCTCAG-TTCCTCCAGTTCCCGCCGGAG
pGBKT7	BD-GR	TGCAT-ATGGCCATGGAGGCCGAATTCTCCCGCCGCTCCGGCGGCGGAGGCG	CAAGGGGTTATGCTAG-TTATGCGGCCGCTCAGTTCCTCCAG-TTCCCGCCGGAG
pCAMBIA1301	OE-OsGRP3	CTGCAGGTCGACTCTAGAG-GATCCATGGCGGCGCCGGATGT	AGAGATGAGTTTCTGCTCGGATCCTCAG-TTCCTCCAGTTCC
